# Atresia Ani Et Recti

**Published:** 1896-04

**Authors:** J. H. Cramer


Operative Treatment of Atresia Ani et Recti, by J. H. Cramer. Some time ago in the district of Ambt-Almelo my assistance was asked by a farmer in the case of four young pigs, which, according to his statement, had no anal opening, and accordingly could make no defecation. On examination I found the four-weeks-old pigs very backward in growth, so that they looked as if they were one to one-and-a-half weeks old. They had bellies as round as a barrel, which gave them a very ridiculous appearance on account of their leanness.

No. 1 had a simple atresia ani, so that after removing a piece of skin the size of a ten-cent piece from the normal situation of the anus, I could press my finger into the rectum. The consequence was that a mass of thin feces was evacuated, certainly a litre, while the apparent plumpness of the animal suddenly vanished. Nos. 2 and 3 were subjected to the same operation with a similar result.

In No. 4, however, there was first an atresia ani and also an atresia recti, so that I could not press my finger into the rectum after removing a piece of skin. At the same time there was present a recto-vaginal fistula, in consequence of which fecal matter had been evacuated by way of the vagina. Recovery I considered impossible here, and the animal was dispatched. The three first-mentioned pigs were very much restored and grew rapidly after some weeks.

Although Möller in his *Operative Surgery* asserts that animals with atresia ani or recti die very soon, if relief by means of an operation is not given, nevertheless these patients remained pretty healthy for twenty-eight days, and continued to live without serious disturbances at least.—*Tijdschr. voor Veeartsenijkunde*, January number.

